# Feasibility and Safety of Percutaneous Cardiac Interventions for Congenital and Acquired Heart Defects in Infants ≤1000 g

**DOI:** 10.3390/children8090826

**Published:** 2021-09-21

**Authors:** Ranjit Philip, Jeffrey Towbin, Neil Tailor, Vijaya Joshi, Jason N. Johnson, Ronak Naik, B. Rush Waller, Shyam Sathanandam

**Affiliations:** 1 Department of Pediatrics, University of Tennessee Health Science Center, Memphis, TN 38163, USA; jtowbin1@uthsc.edu (J.T.); ntailor@uthsc.edu (N.T.); vjoshi@uthsc.edu (V.J.); jjohn315@uthsc.edu (J.N.J.); rnaik2@uthsc.edu (R.N.); brwaller@uthsc.edu (B.R.W.III); ssathan2@uthsc.edu (S.S.); 2 The Heart Institute, Le Bonheur Children’s Hospital, Memphis, TN 38103, USA

**Keywords:** prematurity, cardiac catheterization, low birthweight, congenital heart defects, acquired heart defects, pulmonary valvuloplasty, coarctation stenting, PDA closure

## Abstract

The transcatheter closure of patent ductus arteriosus (TCPC) has been demonstrated to be feasible even in infants weighing ≤1000 g. However, other percutaneous cardiac interventions (PCI) for such small infants born with congenital heart defects (CHD) or acquired heart defects (AHD) have not been well described. The purpose of this study was to describe the feasibility and safety of PCI in infants ≤1000 g. A retrospective review was conducted between June 2015 and May 2021, looking at 148 consecutive PCIs performed on infants weighing ≤1000 g at the time of the procedure. The procedural success rate was 100%. The major adverse event (AE) rate for TCPC was 3%, while there were no major AEs for other PCI. It is feasible to perform PCIs in infants weighing ≤1000 g with CHD and AHD using currently available technologies.

## 1. Introduction

The incidence of extremely low birthweight (ELBW) infants, defined as a birthweight of less than 1000 g, continues to increase [1]. These infants are usually the youngest preterm newborns, gestationally less than 27 weeks. The morbidity and mortality rate for ELBW infants is high [2]. Therefore, if ELBW infants are born with congenital heart defects (CHD), or have acquired heart defects (AHD), operations are deferred except for a patent ductus arteriosus (PDA), where surgical ligations were performed [3]. However, with the advent of transcatheter PDA closure (TCPC) [4], interventional cardiologists are encouraged to perform percutaneous cardiac interventions (PCI) for CHD and AHDs, as there are no surgical alternatives. Infants weighing less than 2 kg are considered to have higher adverse events (AEs) even after adjusting for the procedure risk and their hemodynamic susceptibility [5]. The apprehension regarding PCI in ELBW infants is due to the fear of increased procedure-related AEs, technical challenges, lack of suitable devices, and the overall care required to transport and perform a catheter-based procedure in these fragile infants [6]. With the new technology and device development in the armament of the interventionalist, PCI is being offered in a selective manner in this tenuous cohort of infants [7,8,9,10,11,12,13,14,15,16]. Additionally, improved real-time ultrasound imaging [17,18,19] and specialized transport methods for ELBW infants [20] have helped to overcome some of the challenges. We have previously described our experience with 100 consecutive percutaneous patent ductus arteriosus (PDA) closures in infants ≤1000 g and shown its safety and feasibility [21]. We hypothesize that, with the establishment of an institutional programmatic approach to ELBW infants [22], other non-PDA PCIs in infants ≤1000 g is feasible and safe. In this article, we describe our single-center experience of performing PCI in ELBW infants.

## 2. Materials and Methods

### 2.1. Study Design

A retrospective review of PCIs performed on infants weighing ≤1000 g at the time of the procedure was conducted between June 2015 and May 2021. The approval of the Institutional Review Board from the University of Tennessee Health Science Center was obtained for this retrospective study. 

### 2.2. Data Collection

Demographic and baseline characteristics were obtained including gestational age, procedural age, birthweight and procedural weight. The indication and type of procedures performed were grouped. The primary outcome measures were the procedure success rate, and rates of major and minor AEs. The procedure’s success rate was determined by the proportion of patients in whom the intended intervention was performed, irrespective of the outcome. Major AE was defined as a hemodynamic compromise secondary to the PCI, resulting in mortality or necessitating an additional procedure. Minor AE was defined as any adverse event that did not require additional treatment

### 2.3. Data Analysis

As this is a descriptive study, no statistical analysis was performed. Data were summarized using mean ± SD for numeric data according to the valid normality assumption. The highest and lowest values are also presented to describe the range of values. Categorical variables were reported as a frequency or percent. 

## 3. Results

Twelve different types of procedures were performed by PCI. The total number of procedures were 148, performed on 131 infants weighing ≤1000 g in the cardiac catheterization laboratory. These included TCPC, pulmonary valvuloplasty, foreign body retrieval, pericardiocentesis, wire perforation of the pulmonary valve to treat pulmonary atresia, balloon atrial septostomy, inter-atrial stent placement following radiofrequency perforation in a patient with hypoplastic left heart syndrome (HLHS) with an intact atrial septum, coarctation of the aorta stent placement, ductal stent placement, pulmonary artery balloon angioplasty, and placement of pulmonary artery flow reducers. The number of procedures performed is listed in Table 1.

For the purpose of this article, the 109 TCPC procedures performed will not be discussed in detail, as they have previously been described [16,21]. Among the non-TCPC PCIs, the median weight and age at the time of procedure were 900 g (490–1000) and 28 days (1–52), respectively. The mean birthweight was 750 g ± 172 and the mean gestational age was 25 weeks ± 1 week, reiterating that all infants were ELBW and extremely preterm (less than 28 weeks). The other demographics and baseline characteristics are listed in Table 2.

**Figure 1:**
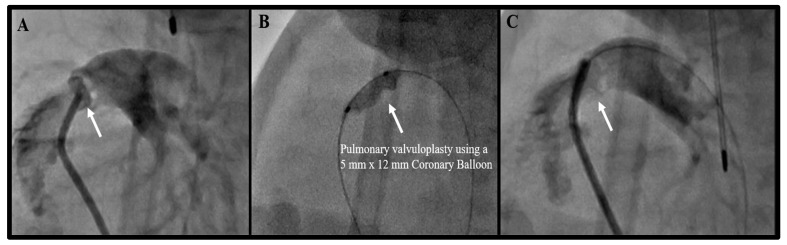
Angiograms of a Pulmonary Valvuloplasty in a 900-gram Infant. (**A**) Angiogram showing a dysplastic, thickened pulmonary valve. (**B**) Balloon angioplasty of the pulmonary valve with an obvious “waist.” (**C**) Post-balloon angioplasty angiogram of the pulmonary valve.

Excluding TCPC, the most common interventions were pulmonary valvuloplasty (Figure 1). Among the ELBW infants that underwent pulmonary valvuloplasty, only one had the procedure without any other associated interventions. Six ELBW infants underwent balloon pulmonary valvuloplasty concurrent with TCPC, while two were balloon pulmonary valvuloplasty, following wire perforation of a plate-like pulmonary valve atresia. The balloons used were semi-compliant coronary artery balloons, typically 5 mm in diameter by a 12 mm in length. The shorter-length balloon was preferred to avoid inadvertent injury to the tricuspid valve. All patients except one required a repeat pulmonary valvuloplasty before hospital discharge. None of the re-interventions are included, as the infants were over 1000 g at that time. One of the pulmonary atresia infants that had wire perforation followed by balloon valvuloplasty eventually underwent another procedure for ductal stenting, as the child continued to be hypoxic. However, following this procedure, the child developed pulmonary over-circulation, necessitating the placement of a pulmonary flow reducer, in the form of a modified, fenestrated microvascular plug (Medtronic Inc. Minneapolis, MN, USA), within the ductal stent. This ELBW infant underwent a total of four procedures, all below a weight of 1000 g, which were counted as four separate procedures.

**Figure 2:**
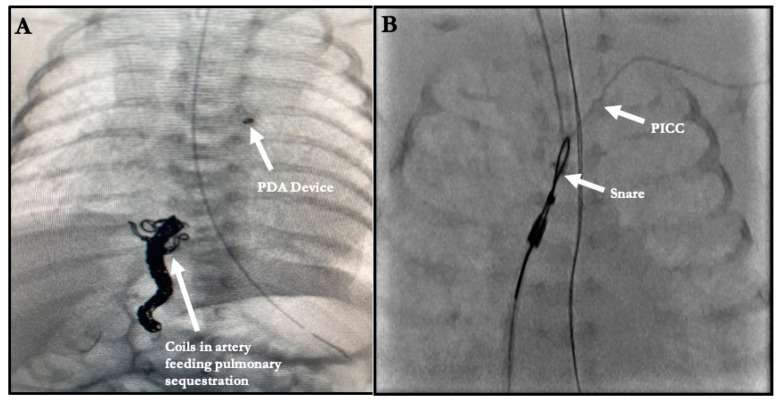
(**A**) Patient in whom the afferent artery to the sequestrated right lower lobe was coil embolized through the PDA with eventual device closure of the PDA. (**B**) Foreign Body Retrieval. Retrieval of a retained peripherally inserted central catheter tip.

Among the TCPC infants, one infant underwent coiling of an afferent artery from the celiac trunk off the descending aorta to a sequestrated lobe of the right lung by accessing a catheter via a femoral venous approach across the PDA. Once the coiling was successfully completed, TCPC was performed (Figure 2A). Similarly, the other patient that underwent coiling of an afferent artery from the suprarenal branch of the descending aorta to a sequestrated right lower lobe had the same approach, from the femoral vein across a PDA, in order to access the culprit vessel. However, in this patient, the PDA was left for eventual spontaneous closure. This patient eventually developed left pulmonary vein stenoses, requiring stent implantations; however, this occurred at a later date when the child was over 1000 g in weight and this procedure was not counted.

Among the nine foreign-body retrievals, two followed TCPC. Both developed left pulmonary artery (LPA) stenosis following device deployment. Both were retrieved with a 5 mm goose neck snare. In one patient, the PDA closed spontaneously and did not require a second device, while in the other, a different device was used to close the PDA. The other seven foreign-body retrievals were broken or misplaced central venous catheters. All these were performed using 5 mm goose neck snares, with a long, 4-French sheath to completely capture the foreign body (Figure 2B).

**Figure 3:**
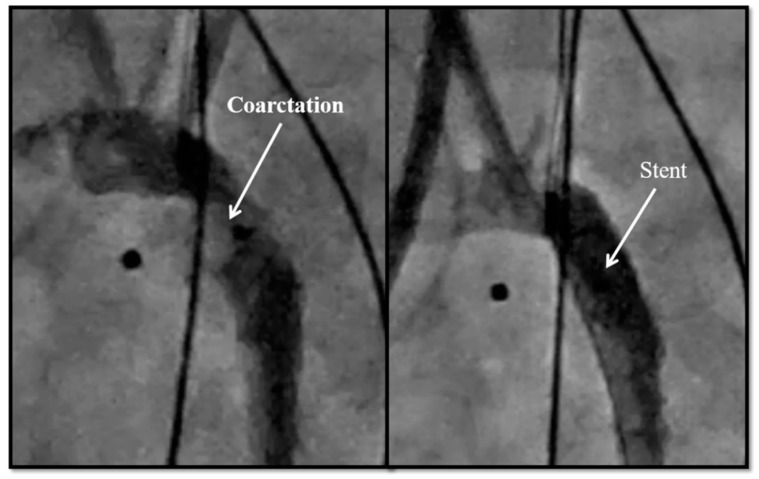
Aortic arch obstruction and subsequent stent placement in a 900 g infant.

All the listed procedures were performed via a femoral venous access-only approach including the two ductal stenting cases, except for the coarctation stents (Figure 3), which were performed via the carotid artery approach. This was accomplished via a surgical cutdown in all except for one, in whom the carotid artery was accessed percutaneously. Of the six coarctation stents, two acquired secondary coarctations to TCPC, where the device caused the stenosis. In both these cases, retrieval attempts were not made, and we proceeded straight to stent implantation. One patient eventually died of pneumonia. The other patient required stent re-dilation at one year of age. All coarctation stents were pre-mounted coronary artery stents. The first four patients had drug-eluting stents, with two patients developing blood stream infections at a later date. It was unclear whether the infection was secondary to the immunosuppression from the drug. Therefore, for the last two patients in this series that required coarctation stenting, bare-metal stents were used instead. Overall, three patients did not survive past a few months, while all three survivors had stent re-dilation at least once, with the eventual goal of stent unzipping [23,24] in the future. 

**Figure 4:**
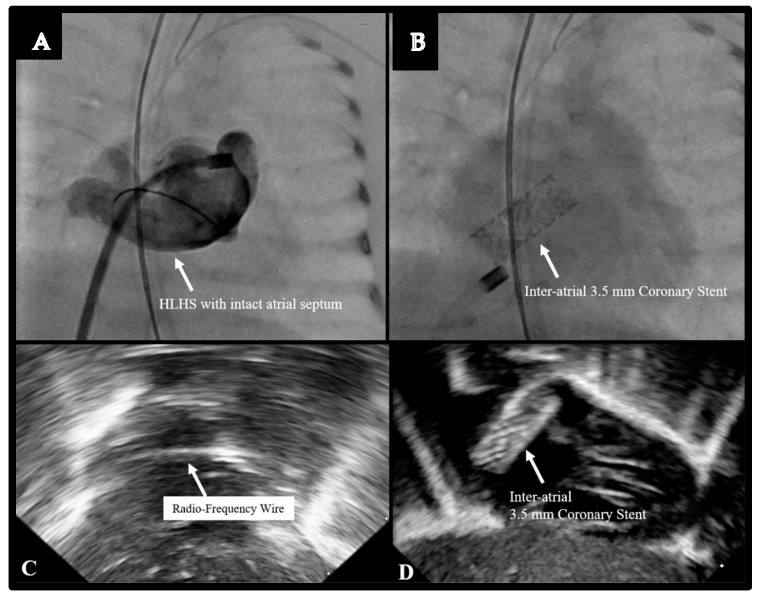
Inter-atrial stent placement in an 890 g infant with Hypoplastic Left Heart Syndrome with an Intact Atrial Septum. (**A**) Angiogram of the intact atrial septum. (**B**) Placement of a coronary stent across the atrial septum. (**C**) Echocardiographic image of the radio-frequency wire across the atrial septum. (**D**) Echocardiographic image of the coronary stent.

The use of trans-thoracic echo (TTE) guidance was key for all TCPC procedures, as well as for the balloon atrial septostomy in a patient born with d-transposition of the great arteries (d-TGA), and in another patient born with hypoplastic left heart syndrome (HLHS) with atresia of the mitral and aortic valves along with an intact atrial septum. In this 800 g infant, who was severely cyanotic and acidotic, TTE-guided radiofrequency perforation of the atrial septum was performed, followed by the implantation of a 3.5 mm coronary stent across the atrial septum (Figure 4). This was accomplished entirely under TTE guidance. Although the patient stabilized after this procedure, as well as another procedure for ductal stenting via a femoral venous approach, we eventually withdrew support at 30 days of age, given the poor outcome possibility for an 800 g baby with HLHS. 

The overall procedure success rate was 100%. At the latest follow-up, the survival rate was 87.8% (*n* = 115/131). Ten of the TCPC patients did not survive at latest follow-up. However, only one was a procedure-related mortality, while the others were complications of prematurity. One such mortality was a patient who also underwent balloon pulmonary valvuloplasty during TCPC. This patient required three further balloon dilations of the valve before dying of unrelated causes at 18 months of age. Overall, there were three patient mortalities unrelated to the procedure among those that underwent balloon pulmonary valvuloplasty; two had TCPC as well. As mentioned earlier, three of the coarctation stent patients were not alive at latest follow-up, as well as the patient with HLHS who had inter-atrial stent and ductal stent implantation, and a d-TGA patient that underwent septostomy. The other two mortalities included a patient that underwent balloon pulmonary angioplasty and one patient that required an emergency pericardial drain. The first patient had an unknown syndrome with severely hypoplastic branch pulmonary arteries. This patient was severely hypoxic at birth and the balloon angioplasty was offered to prevent a near certain mortality. A 2 mm coronary balloon was used to dilate the proximal right and left pulmonary arteries. However, during the procedure, it was noted that there were stenoses in every lobar, segmental and sub-segmental branches, which were too small for any interventions to be performed. Therefore, soon after the procedure, the decision was made to withdraw support. The other patient that had the pericardial drain was the smallest patient in this cohort, born at 22 weeks’ gestation, and weighed 440 g when the pericardial drain was placed to treat a central line perforation. This patient died at 2 weeks of age from unrelated complications of prematurity. 

Three major AEs were recorded during TCPC. One was a procedural mortality from laceration of the inferior vena cava. The other two were coarctations that required stent implantation. There were also three minor AEs for TCPC. Two LPA stenosis required device retrieval and one had a pericardial effusion that required drainage. There were no major AEs for the non-TCPC procedures. There was one minor AE during one of the coarctation stenting procedures, secondary to TCPC. An intimal flap was raised in the descending aorta by a 0.014” coronary wire. This flap was not visualized at the end of the procedure. Table 3 summarizes the complication rate and current fate of each patient based on the type of procedure they received. 

## 4. Discussion

This single-center experience is a good reflection of the current capabilities of a cardiac catheterization laboratory in this cohort of ELBW preterm infants, where surgical options are either contraindicated or considered high-risk. In this series, the procedure success rate was 100%, while the overall major AE rate for this entire cohort of 148 patients that underwent PCI is 2% and the minor AE rate is 2.7%, with almost no AEs for the non-TCPC PCIs. These results are very encouraging. Many studies evaluate extreme gestational age rather than birthweight and although there is an overlap between the two, the distinction is important when evaluating procedure-related outcomes. With the advances in technological interventions, rate of survival to discharge continues to improve [25]. This makes it imperative to continue to offer interventions that can be safely performed on this population. With newer devices such as the Amplatzer Piccolo Occluder (Abbott, Plymouth, MA, USA), manufactured specifically for infants as small as 700 g [11], access to microcatheters, use of less stiff catheters such as the glide catheter (Terumo, Japan), and thin-walled sheaths such as the Prelude Ideal sheath (Merit Medical, South Jordan, UT, USA) the confidence of pediatric interventional cardiologists to perform procedures on babies that weigh less than 1000 g has greatly improved. As advocates for these fragile infants, interventionalists and care providers must be cognizant that the ability to safely perform a procedure should not be an indication for the procedure. Strict hemodynamic criteria and clear indications for elective procedures such as TCPC are essential, even after failed medical therapy. Procedures for HLHS with an intact atrial septum in infants less than 1 kg have very poor survival rates and carry a high risk for mortality with definitive surgery. Although an emergency life-saving procedure, radiofrequency perforation for HLHS with an intact atrial septum with an inter-atrial stent is also a high-risk procedure. However, it can be performed with the intent of providing palliation for a future definitive surgery. The decision in instances where the long-term outcome may not be favorable must be made with extreme care and thought. 

The growing experience [7,8,9,10,11,16,21] and learning curve with a protocolized TCPC program [22] has possibly helped increase comfort levels not only for the interventionalist performing the procedure but also for the neonatologist referring the infant and the anesthesiologist caring for the infant during the procedure. Paying attention to detail from the transport from the NICU [20], to the ventilatory support en-route and in the catheterization laboratory, as well as vascular access in these tiny, fragile infants, is of paramount importance. The strict maintenance of infant thermal stability and the cognizant effort to provide efficient transport and procedure management goes a long way in maintaining infant hemodynamics by avoiding unnecessary prolongation of the procedure. In certain procedures, such as ductal and atrial stenting, similar to TCPC, precise and quick-thinking echocardiographic guidance [17,18,19] helps the interventionalist with the procedure-time in addition to confirming a successful procedural outcome. Avoiding femoral arterial access in these small infants is of the utmost importance to prevent access-related complications [26,27,28]. Similarly, the use of ultrasound for femoral venous access is important to avoid inadvertent injury to the femoral artery. Ultrasound guidance also improves the success rate for vascular access, as well as reducing the overall procedure time [27]. All the listed procedures were performed via a femoral-venous-access-only approach, including the two ductal stenting cases and the two arterial coiling cases, with the exception of the coarctation stents, which were performed via the carotid artery approach. The growing experience with percutaneous carotid artery access for other neonatal interventions makes this an attractive alternative. Ultrasound guidance is absolutely essential for this purpose. 

The management of complications related to TCPC [29] such as aortic coarctation and pericardial effusions has inadvertently improved the skill set of the interventionalist and enlightened the medical community to the feasibility of performing procedures such as pericardiocentesis and aortic arch stenting in this cohort. The familiarity and accessibility of these miniature devices and delivery systems in TCPC has enabled the new application of these devices as pulmonary artery flow reducers for congenital heart diseases (ventricular septal defects, hypoplastic left heart syndrome, truncus arteriosus) providing a palliative treatment option until the infants grow older and become eligible for definitive surgical therapy [30,31]. 

There are limitations to this study. There is a possibility of selection bias. Only the best candidates could have been offered these interventions. However, we can state that we have never turned down any patient for TCPC. There were no surgical PDA ligations during this time period. Therefore, the selection bias is limited. Similarly, for other procedures, as there were no good surgical or medical alternatives, it was difficult to have selection bias. Moreover, many of these procedures were performed knowing that the alternative was certain mortality. Although the procedure was performed, we decided to withdraw support in at least three situations, understanding the poor prognosis for these patients. One may argue the futility of these procedures. However, a multi-disciplinary approach involving palliative care, neonatology, cardiology and shared decision-making with the family is of paramount importance. The other limitation of this study is the lack of a comparison group. This is, again, due to no alternatives being available. 

## 5. Conclusions

It is feasible to perform complex, percutaneous, transcatheter interventions for congenital and acquired heart defects in infants weighing ≤1000 g using currently available technologies. There is a learning curve with these interventions, with most AEs happening earlier in the experience. Extreme care must be taken while performing interventions in such fragile human beings. Further miniaturization of equipment will facilitate better outcomes. 

## Figures and Tables

**Table 1:** Type and Number of Procedures.

Procedure Type	*n*
1. Transcatheter PDA Closure	109
2. Pulmonary Valvuloplasty	9
3. Foreign Body Retrieval	8
4. Pericardiocentesis	6
5. Coarctation of the aorta stent	6
6. Ductal Stent	2
7. Pulmonary valve perforation for pulmonary atresia	2
8. Coiling of artery to pulmonary sequestration	2
9. Balloon atrial septostomy	1
10. Radiofrequency perforation for HLHS with intact atrial septum with inter-atrial stent placement	1
11. Pulmonary artery balloon angioplasty	1
12. Pulmonary artery flow reducer	1

**Table 2:** Demographics and Baseline Characteristics.

	Non-TCPC Interventions (*n* = 39)
Procedure Weight (g)	
Mean ± SD	838 ± 169
Median (Range)	900 (440–1000)
Procedure Age (days)	
Mean ± SD	24 ± 12
Median (Range)	28 (1–52)
Gestational Age (weeks)	
Mean ± SD	25 ± 1
Median (Range)	25 (22–28)
Birth Weight (g)	
Mean ± SD	750 ± 172
Median (Range)	700 (400–980)
Sex, Male	54% (21)

**Table 3:** Adverse Events (AE) and Follow-Up (F/U).

Procedure Type	*n*	Major AE *n* (%)	Minor AE *n* (%)	Survival at Latest F/U (Median = 3 Years)
1. Transcatheter PDA Closure (TCPC) *^^‡¶œ^	109	3	3	99
2. Pulmonary Valvuloplasty *^∂€^	9	0	0	6
3. Foreign Body Retrieval ^^^	8	0	0	8
4. Pericardiocentesis ^‡^	6	0	0	5
5. Coarctation of the aorta stent	6	0	1	3
6. Ductal Stent ^∂†^	2	0	0	1
7. Pulmonary valve perforation for pulmonary atresia ^€∂^	2	0	0	1
8. Coiling of artery to pulmonary sequestration ^œ^	2	0	0	2
9. Balloon atrial septostomy	1	0	0	0
10. Radiofrequency perforation for HLHS with intact atrial septum with inter-atrial stent placement ^†^	1	0	0	0
11. Pulmonary artery balloon angioplasty	1	0	0	0
12. Pulmonary artery flow reducer ^∂^	1	0	0	1
Overall Total	148	3 (2%)	4 (2.7%)	87.8% (115/131)

* 6 patients underwent TCPC and pulmonary valvuloplasty with 2 non-survivors at latest F/U. ^^^ 2 patients underwent TCPC and Foreign Body Retrieval. ^‡^ 1 patient underwent TCPC and pericardiocentesis. ^¶^ 2 patients underwent TCPC and coarctation of the aorta stent with 1 non-survivor at latest F/U. ^œ^ 1 patient underwent TCPC and coiling of artery to pulmonary sequestration. ^€^ 1 patient underwent pulmonary valve perforation and pulmonary valvuloplasty. ^†^ 1 HLHS patient underwent atrial stent and ductal stent who was a non-survivor at latest F/U. ^∂^ 1 patient underwent pulmonary valve perforation, pulmonary valvuloplasty, ductal stent and pulmonary artery flow reducer implantation

## Data Availability

Data supporting reported results can be provided on request.
